# Interferon-primed immune landscapes predict immune-related adverse events during immune checkpoint inhibitor therapy

**DOI:** 10.3389/fcell.2026.1798845

**Published:** 2026-05-08

**Authors:** Juanjuan Kang, Rong Huang, Yumei Chen, Yang Yu, Xinyu Li, Xi Yong, Guangyu Ao, Qin Yang

**Affiliations:** 1 State Key Laboratory of Southwestern Chinese Medicine Resources, and Innovative Institute of Chinese Medicine and Pharmacy, Chengdu University of Traditional Chinese Medicine, Chengdu, Sichuan, China; 2 School of Pharmacy, North Sichuan Medical College, Nanchong, China; 3 Affiliated Hospital of Clinical School of Medicine, North Sichuan Medical College, Institute of Neurological Diseases, Nanchong, China; 4 School of Intelligent Medicine, Chengdu University of Traditional Chinese Medicine, Chengdu, China; 5 Department of Vascular Surgery, Affiliated Hospital of North Sichuan Medical College, Nanchong, China; 6 Sichuan Branch of National Clinical Research Center for Digestive Diseases, Nanchong, China; 7 Chengdu Integrated TCM&Western Medicine Hospital, Chengdu, China; 8 Department of Infectious Diseases, Affiliated Hospital of North Sichuan Medical College, Nanchong, China

**Keywords:** drug repurposing, immune checkpoint inhibitor, immune-related adverse events, interferon signaling, machine learning

## Abstract

**Background:**

Immune checkpoint inhibitors (ICIs) have transformed cancer therapy but frequently induce immune-related adverse events (irAEs), which can disrupt treatment and worsen outcomes. The mechanisms predisposing certain individuals to irAEs remain unclear, and reliable strategies for early prediction and prevention are urgently needed.

**Methods:**

We analyzed pre-treatment peripheral blood mononuclear cell (PBMC) transcriptomic data from 88 patients receiving ICIs, including 22 who subsequently developed irAEs. Immune infiltration signatures were used to build machine learning models with SHAP-based interpretability. Immune-related differentially expressed genes were then incorporated into the Chemical-Induced Gene Signature (CIGS) framework to predict candidate reversal compounds. Selected compounds were further evaluated in Jurkat T cells to experimentally validate their effects on interferon-γ signaling and underlying mechanisms.

**Results:**

Baseline immune infiltration patterns showed strong predictive value for subsequent irAE development, with the Random Forest model achieving the best performance (AUC = 0.97). SHAP analysis revealed that activated T-cell and NK-cell signatures were dominant predictors, indicating a pre-existing immune-primed state in irAE-prone individuals. Single-cell analysis identified two irAE-enriched myeloid clusters with lung-associated inflammatory features, suggesting baseline myeloid priming. T cells and NK cells from irAE samples exhibited marked upregulation of interferon-stimulated genes and strong enrichment of type I and type II interferon pathways. Perturbation-based screening identified multiple compounds capable of reversing these interferon-amplified signatures, and *in vitro* experiments demonstrated that alpinetin and momelotinib suppress interferon-γ signaling through distinct STAT1-and JAK–STAT–dependent mechanisms.

**Conclusion:**

irAEs may arise from the convergence of pre-existing myeloid inflammation and interferon-driven lymphocyte activation before therapy. Our study provides a predictive framework for identifying high-risk patients and highlights mechanistically grounded compounds for potential irAE mitigation.

## 1 Introduction

Immune checkpoint inhibitors (ICIs) have transformed cancer therapy by reinvigorating antitumor immunity and improving survival across multiple solid tumors. However, ICIs can also trigger immune-related adverse events (irAEs), a diverse spectrum of inflammatory toxicities affecting nearly all organ systems and ranging from mild to life-threatening manifestations (Postow et al., 2018; Remond et al., 2018). These toxicities have emerged as a major limitation to treatment continuity and patient prognosis, and their unpredictable nature underscores the urgent need for mechanistic insight and reliable early-warning biomarkers (Liu et al., 2025; Puzanov et al., 2017; Usama et al., 2025).

Accumulating evidence indicates that irAEs are associated with excessive or misdirected immune activation driven by ICI therapy, characterized by dysregulated cytokine signaling, heightened type I and type II interferon responses, and aberrant activation of T cells, NK cells, monocytes, and macrophages (Chen et al., 2023; Kanaji et al., 2025). Recent single-cell studies further reveal distinct immune cell states associated with irAE onset, including interferon-amplified monocyte programs and pro-inflammatory T-cell activation signatures (Kanaji et al., 2025; Sun et al., 2025). Despite these advances, the cell-type specific mechanisms that predispose certain patients to irAEs remain incompletely defined. Moreover, although the occurrence of irAEs has been linked to improved antitumor efficacy in several cancers (Bastacky et al., 2021), prediction of irAE risk and effective pharmacological prevention strategies remain unmet clinical needs.

Although previous studies have characterized immune remodeling in the tumor microenvironment, affected tissues, or samples collected after toxicity onset, far fewer have investigated whether systemic immune signatures present before treatment can identify patients predisposed to irAEs. To address these challenges, we carried out a multi-dimensional investigation integrating bulk immune-activity profiling, single-cell transcriptomics and immune-lineage–resolved analyses. We first quantified baseline immune infiltration in pre-treatment PBMCs from ICI-treated patients and developed machine learning models to predict irAE occurrence, supported by SHAP-based interpretability. We then analyzed single-cell RNA sequencing (scRNA-seq) data from non-small cell lung cancer patients to identify cellular remodeling and inflammatory states enriched in individuals predisposed to irAEs. By focusing on NK and T lymphocytes, the key mediators of irAE pathology, we characterized cell-type–specific interferon activation and effector programs associated with irAE susceptibility. Finally, using chemical-induced gene-signature resources, we screened for candidate compounds capable of reversing irAE-associated transcriptional programs, providing a basis for potential preventive interventions.

Collectively, this study provides an integrated framework for irAE prediction, mechanistic dissection at single-cell resolution, identification of pathogenic immune programs, and rational discovery of potential agents for irAE prevention. Our findings deepen the understanding of ICI-induced immune dysregulation and offer translational insights toward mitigating irAE-associated morbidity without compromising antitumor efficacy.

## 2 Materials and methods

### 2.1 Sample collection

Peripheral blood mononuclear cell (PBMC) samples from patients receiving ICI therapy were retrieved from the Gene Expression Omnibus (GEO) database. To ensure accurate irAE classification, we included only datasets with: (i) PBMC samples collected prior to the initiation of ICI therapy, and (ii) explicit annotation distinguishing irAE from non-irAE cases. According to these criteria, three datasets were incorporated: GSE287540 (bulk RNA-seq) (Ji et al., 2025), GSE287541 (scRNA-seq) (Ji et al., 2025), and GSE285888 (scRNA-seq) (Kim et al., 2025), covering melanoma, renal cell carcinoma, and non-small cell lung cancer (NSCLC). A total of 88 patients were included (22 irAE; 66 non-irAE). All samples were collected before the first ICI treatment, ensuring that transcriptomic signatures reflected baseline immune states prior to irAE onset (Table 1). Because this study was based on publicly available matrices rather than raw FASTQ files, no *de novo* read alignment was performed in the present analysis. For scRNA-seq data, raw count matrices provided by GEO were imported into Seurat for downstream quality control, normalization, clustering, and differential expression analysis.

**TABLE 1:** Summary of the pre-treatment PBMC transcriptomic datasets included in this study.

Dataset	Cancer type	Transcriptomic data type	irAE cases (n)	Non-irAE cases(n)
GSE287541	Melanoma	scRNA-seq	3	6
​	Renal	scRNA-seq	1	3
GSE285888	NSCLC	scRNA-seq	7	20
GSE287540	Unknown	Bulk RNA-seq	11	37
Total	​	​	22	66

### 2.2 irAE prediction model construction

To integrate bulk and single-cell datasets, scRNA-seq samples were aggregated into pseudo-bulk matrices by summing gene-level counts across all cells in each sample. Immune cell activity was quantified using single-sample Gene Set Enrichment Analysis (ssGSEA). Marker gene sets corresponding to 28 immune cell types were obtained from TISIDB and used to infer immune infiltration patterns for each sample.

Using the ssGSEA-derived immune signatures, six machine learning algorithms, XGBoost, Support Vector Machine (SVM), Random Forest (RF), k-Nearest Neighbors (KNN), Gaussian Naïve Bayes (GNB), and LightGBM, were implemented to build irAE prediction models (Guo et al., 2025; Kang et al., 2020; Tang et al., 2026). All models were trained and tuned using identical feature sets to ensure comparability.

### 2.3 irAE prediction model evaluation

Given the limited cohort size, predictive performance was evaluated using five-fold cross-validation (Pham et al., 2024; Nguyen et al., 2025). For each fold, models were trained on 80% of samples and validated on the remaining 20%. Model performance was assessed using sensitivity (Sn), specificity (Sp), accuracy (Acc), F1-score, Matthew’s correlation coefficient (MCC) (Equations 1–5), and area under the ROC curve (AUC) (Zhang et al., 2025; Zhu et al., 2023; Zeng et al., 2025). The evaluation metrics were calculated as follows:

$$\text{Sensitivity }\left(\text{Sn}\right)=\frac{TP}{TP+FN}$$ (1)

$$\text{Specificity }\left(\text{Sp}\right)=\frac{TN}{TN+FP}$$ (2)

$$\text{Accuracy }\left(\text{Acc}\right)=\frac{TP+TN}{TP+TN+FP+FN}$$ (3)

$$F1‐\text{score}=\frac{2\times TP}{2\times TP+FP+FN}$$ (4)

$$\text{MCC}=\frac{TP\times TN-FP\times FN}{\sqrt{\left(TP+FP\right)\left(TP+FN\right)\left(TN+FP\right)\left(TN+FN\right)}}$$ (5)

AUC values were derived from ROC curves to evaluate overall discriminative power. All metrics were averaged across folds to ensure robustness (Tran et al., 2025; Kang et al., 2019).

### 2.4 Interpretable analysis of the prediction model based on SHAP

To interpret feature contributions to irAE prediction, we applied SHapley Additive exPlanations (SHAP). SHAP values quantify the marginal impact of each immune feature on model output based on cooperative game theory (Yang et al., 2023). For the best-performing classifier, SHAP values were computed using TreeExplainer (for XGBoost, RF, LightGBM) or KernelExplainer (for SVM, KNN, GNB). A SHAP summary beeswarm plot was generated to visualize global feature importance and the directionality of feature effects, enabling identification of immune signatures most strongly associated with irAE susceptibility.

### 2.5 scRNA-seq analysis

ScRNA-seq data from non–small cell lung cancer (NSCLC) patients were obtained from the GEO dataset GSE285888. Raw count matrices were imported into Seurat (R package) for downstream analysis. Cells with fewer than 200 detected genes, more than 6000 detected genes, or mitochondrial gene proportions exceeding 15% were removed as low-quality cells or potential doublets. Gene expression values were normalized and log-transformed, and highly variable genes were identified and used for principal component analysis (PCA). The top principal components were then used to construct a shared nearest neighbor (SNN) graph, followed by unsupervised clustering (Louvain algorithm) and visualization with Uniform Manifold Approximation and Projection (UMAP). Cluster-level marker genes were identified using FindAllMarkers (Wilcoxon test, only positive markers, min.pct = 0.25, logfc.threshold = 0.25).

After clustering and UMAP embedding, cell type annotation was performed by integrating automated and marker-based approaches. First, we applied the SingleR package with celldex datasets to obtain an initial automatic annotation for each cluster. To increase robustness, these labels were further cross-validated and refined using curated marker gene resources from CellMarker 2.0 (Hu et al., 2023) and PanglaoDB (Franzen et al., 2019), ensuring consistency with known immune and stromal cell signatures in peripheral blood and NSCLC-related tissues.

Based on the final annotation, cells assigned to T-cell and NK-cell lineages were subset for downstream analyses. Within each lineage, differential gene expression between irAE and non-irAE groups was assessed using Seurat’s FindMarkers function (Wilcoxon rank-sum test) with multiple-testing correction (Benjamini–Hochberg method). The resulting T-cell and NK-cell specific transcriptional changes were used to characterize immune remodeling associated with irAE development.

### 2.6 Enrichment analysis

For irAE-enriched clusters, high-expressing marker genes were subjected to cell signature enrichment using Metascape database (Zhou et al., 2019). To characterize pathway perturbations within specific immune lineages, GO enrichment analysis was performed on genes upregulated in irAE samples. Additionally, Gene Set Enrichment Analysis (GSEA) was applied to ranked DEGs from T-cell and NK-cell subsets, using immune-related gene sets obtained from ImmPort (Bhattacharya et al., 2018) to quantify activation of cytokine signaling, lymphocyte activation, interferon responses, and inflammatory pathways.

### 2.7 Candidate drug screening

To prioritize compounds capable of modulating irAE-associated transcriptional programs, we performed perturbation analysis using the Chemical-Induced Gene Signature (CIGS) database (Xiang et al., 2025). CIGS includes expression signatures for 13,221 compounds (11,356 MCE bioactive molecules; 1,865 TCM compounds) across two human cell lines (MDA-MB-231 and HEK293T), comprising 93,644 perturbations and >319 million gene-expression events.

irAE-upregulated immune genes were intersected with curated immune-related genes to generate irAE-specific perturbation signatures. These were compared against CIGS using the Zhang score algorithm to assess similarity or reversal potential. Compounds with positive Zhang scores were considered mimickers, whereas those with negative Zhang scores were designated reversal candidates, reflecting their potential to counteract irAE-associated immune activation. Compounds were ranked according to the direction and magnitude of their Zhang scores. Candidate compounds for downstream validation were further prioritized based on their predicted reversal potential, biological relevance to interferon-related or inflammatory signaling, literature support, and experimental feasibility.

### 2.8 CCK-8 assay

The Jurkat T-cell line used in this study was obtained from Immocell Biotechnology Co., Ltd. (Xiamen, China) and maintained under standard culture conditions. Briefly, Jurkat cells were cultured in RPMI-1640 medium supplemented with 10% fetal bovine serum (FBS) and 1% penicillin–streptomycin at 37 °C in a humidified incubator with 5% CO_2_. For the cytotoxicity assay, cells were seeded into 96-well plates at a density of 1 × 10^4^ cells per well. Cells were treated with Alpinetin, Mianserin, or Momelotinib sulfate using an eight-point concentration gradient (0, 0.1, 1, 5, 10, 20, 50, and 100 μM). After 24 h of compound treatment, Cell Counting Kit-8 (CCK-8) reagent was added to each well according to the manufacturer’s instructions, followed by incubation at 37 °C for 1–2 h. Absorbance was measured at 450 nm using a microplate reader. Cell viability was calculated as a percentage relative to the vehicle control. All experiments were performed in triplicate (n = 3).

### 2.9 Western blot analysis

Jurkat cells were treated with Alpinetin, Mianserin, or Momelotinib sulfate at three concentrations. Alpinetin and Mianserin were used at 1, 5, and 20 μM, whereas Momelotinib sulfate was applied at 1, 5, and 10 μM. Cells were pre-treated with each compound for 1 h, followed by stimulation with recombinant human interferon-γ (IFN-γ, 50 ng/mL). After 24 h of IFN-γ stimulation, cells were harvested, washed twice with cold PBS, and lysed in ice-cold RIPA buffer supplemented with protease and phosphatase inhibitor cocktails. Cell lysates were clarified by centrifugation at 4 °C, and protein concentrations were determined using a BCA assay. Equal amounts of protein were resolved by SDS-PAGE and transferred onto PVDF membranes. Membranes were blocked with 5% BSA in TBST and incubated overnight at 4 °C with primary antibodies against STAT1 and phosphorylated STAT1, followed by incubation with HRP-conjugated secondary antibodies. Protein bands were visualized using enhanced chemiluminescence (ECL) and quantified by densitometric analysis. Phosphorylated protein levels were normalized to total protein levels, and total protein expression was normalized to GAPDH.

## 3 Results

### 3.1 Immune infiltration–based machine learning models accurately predict irAE occurrence

**FIGURE 1:**
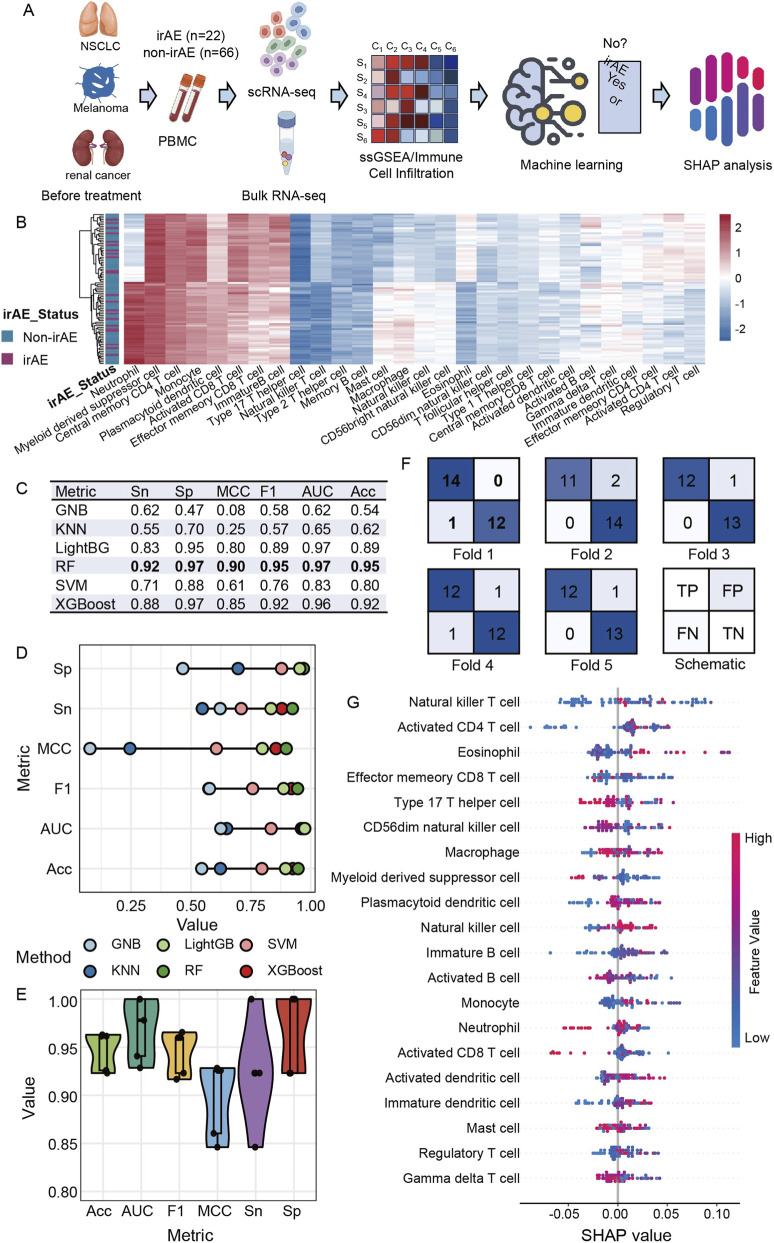
Immune infiltration–based prediction of irAE and interpretation of model features. **(A)** Overview of the analytical workflow. **(B)** Heatmap of ssGSEA-derived immune infiltration patterns in irAE and non-irAE samples. **(C)** Performance metrics of six machine learning classifiers assessed by five-fold cross-validation. **(D)** Comparison of classifier performance across evaluation metrics. **(E)** Violin plot of RF model performance metrics across five-fold cross-validation. **(F)** Confusion matrices of the RF model for each cross-validation fold. **(G)** SHAP global beeswarm plot illustrating the contribution of ssGSEA-derived immune cell signatures to irAE prediction in RF model.

We constructed an integrated analytical workflow combining bulk RNA sequencing and single-cell transcriptomic data to characterize baseline immune profiles associated with irAE development and to build a predictive model (Figure 1A). Peripheral blood mononuclear cell samples collected before the initiation of ICI therapy were obtained from melanoma, renal cell carcinoma and non-small cell lung cancer cohorts, including twenty-two patients who later developed irAEs and sixty-six who did not. Single-cell datasets were aggregated into pseudo-bulk matrices and analyzed alongside bulk RNA sequencing data to obtain comprehensive immune infiltration profiles. Using ssGSEA with twenty-eight immune-cell signatures, we quantified immune cell abundance for each patient and used these features for downstream machine learning analysis. A heatmap of immune infiltration patterns further illustrated the baseline immune landscape across the irAE and non-irAE groups (Figure 1B).

**FIGURE 2:**
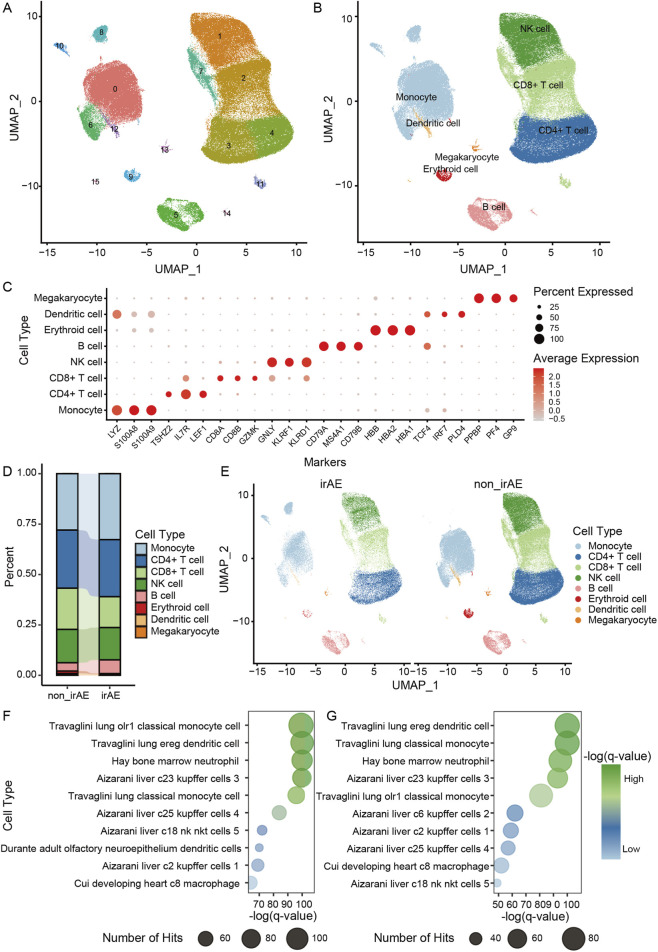
Single-cell landscape of NSCLC PBMCs and identification of irAE-specific immune clusters. **(A)** UMAP visualization of all PBMCs from NSCLC patients. **(B)** Annotation of major immune lineages. **(C)** Dot plot showing expression levels of key canonical markers across annotated immune cell types. **(D)** Comparison of immune cell composition between irAE and non-irAE groups. **(E)** UMAP split view showing the distribution of annotated cell types in irAE versus non-irAE samples. **(F)** Cell-type signature enrichment analysis for cluster 8. **(G)** Cell-type signature enrichment for cluster 10.

Six machine learning classifiers were trained using five-fold cross-validation. The RF model achieved the best performance, with high sensitivity, specificity, MCC, F1-score, AUC (Figures 2C,D). XGBoost and LightGBM also performed well, whereas GNB and KNN showed limited predictive power. To further evaluate model robustness, we summarized the performance metrics of the RF model across the five validation folds using a violin plot, which showed consistently strong predictive performance (Figure 1E). In addition, confusion matrices from each fold demonstrated stable classification accuracy for both irAE and non-irAE samples (Figure 1F). These findings indicated that baseline immune infiltration signatures, even before immunotherapy begins, can robustly distinguish patients who later develop irAEs from those who do not. This observation is consistent with recent reports suggesting that irAEs arise not only from treatment-induced immune activation but also from pre-existing inflammatory predispositions reflected in peripheral immune signatures (Khan et al., 2025).

To interpret the most influential immune features driving prediction, we applied SHAP analysis to the best-performing classifier. The global beeswarm plot (Figure 1G) revealed that levels of natural killer T cells, activated CD4^+^ T cells, effector memory CD8^+^ T cells, type 17 helper T cells and CD56dim natural killer cells were strongly associated with increased irAE risk. These lymphocyte subsets share a common functional hallmark characterized by heightened cytotoxicity and increased responsiveness to type I and type II interferon signaling. This is in agreement with published single-cell studies demonstrating that increased interferon-driven activation of T cells and natural killer cells precedes or accompanies irAE onset (Chen et al., 2023). The convergence of our findings with these external datasets suggests that excessive or primed lymphocyte activation may represent a core immunological precursor of irAE development.

Collectively, these results show that baseline immune infiltration patterns carry strong predictive signals for irAE occurrence, and they highlight activated natural killer cells and T-cell subsets as key cellular drivers of susceptibility. The identification of these signatures before therapy supports the concept that irAEs may arise from a pre-existing, hyper-responsive immune landscape that is further amplified by checkpoint blockade, ultimately leading to pathological inflammation.

### 3.2 Single-cell landscape of NSCLC PBMCs and identification of irAE-specific immune clusters

We next analyzed NSCLC-derived PBMC single-cell transcriptomic data to investigate cell-type–specific alterations associated with irAE development. Unsupervised clustering followed by UMAP projection identified multiple major immune populations, including monocytes, dendritic cells, CD4^+^ T cells, CD8^+^ T cells, natural killer cells, B cells, erythroid cells and megakaryocytes (Figures 2A,B). Canonical marker genes such as LYZ for monocytes, IL7R for CD4^+^ T cells, CD8A for CD8^+^ T cells, GNLY and NKG7 for natural killer cells, and MS4A1 for B cells showed expected expression patterns, confirming the accuracy of the annotations (Figure 2C).

Figure 2D shows the overall distribution of major immune lineages in irAE and non-irAE samples, with no marked differences at the global level. Nevertheless, the subtle variation in lymphocyte and myeloid profiles suggested that deeper inspection at the cluster and transcriptional levels was necessary. Indeed, at higher resolution, two monocyte-derived clusters, cluster eight and cluster 10, were found to be substantially enriched in irAE samples (Figure 2E). The presence of these irAE-specific clusters before treatment suggests that baseline innate immune alterations may influence irAE susceptibility.

To characterize the biological identity of these irAE-associated clusters, we performed cell-type signature enrichment analysis based on their highly expressed genes. Both clusters showed strong enrichment for signatures related to lung classical monocytes, lung dendritic cells and bone marrow derived neutrophils (Figures 2F,G). These transcriptional features indicate that these cells resemble a lung-associated, inflammation-primed myeloid phenotype rather than resting circulating monocytes or dendritic cells.

Collectively, our findings suggest that patients who later develop irAEs may already harbor a pre-existing, lung-associated inflammatory myeloid program in their PBMC compartment prior to ICI therapy. This primed immune state may lower the threshold for checkpoint blockade to trigger excessive myeloid and lymphocyte activation, providing a mechanistic foundation for irAE initiation. These results support the emerging concept that irAEs arise not solely from treatment-induced immune activation but also from baseline immune predisposition detectable before therapy.

### 3.3 T-cell specific transcriptomic analysis reveals pronounced interferon activation and inflammatory pathway upregulation in irAE cases

**FIGURE 3:**
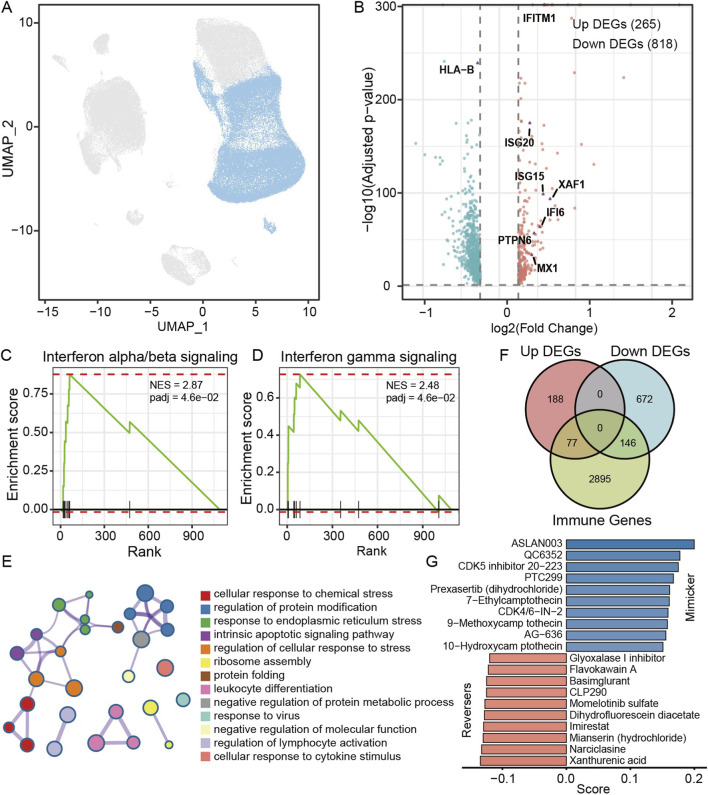
T-cell transcriptional remodeling reveals strong interferon activation and inflammatory pathway upregulation in irAE cases. **(A)** UMAP visualization of all T cells isolated from NSCLC PBMC scRNA-seq data. **(B)** Volcano plot of DEGs between irAE and non-irAE T cells. **(C,D)** Gene Set Enrichment Analysis demonstrating strong enrichment of interferon-α/β signaling (C; NES = 2.87) and interferon-γ signaling (D; NES = 2.48) in irAE T cells. **(E)** GO biological process enrichment based on genes upregulated in irAE T cells. **(F)** Overlap between T-cell DEGs and immune-related gene sets. **(G)** CIGS perturbation analysis identifying compounds predicted to mimic (upper panel) or reverse (lower panel) the irAE-associated T-cell transcriptional state.

To investigate intrinsic T-cell alterations associated with irAE development, we isolated all annotated T cells from the NSCLC scRNA-seq dataset (Figure 3A) and performed differential and pathway-level analyses. A total of 265 genes were upregulated and 818 genes were downregulated in T cells from irAE patients compared to non-irAE controls (Figure 3B).

GSEA showed strong activation of both interferon-α/β signaling and interferon-γ signaling in irAE T cells, with normalized enrichment scores (NESs) of 2.87 and 2.48 respectively (Figures 3C,D). Consistent with the GSEA findings, the majority of genes contributing to the interferon α/β signaling pathway appeared among the upregulated differentially expressed genes in irAE T cells (Figure 3B). These interferon-stimulated genes, including ISG15, MX1, IFI6, IFITM1, and XAF1, form the core transcriptional backbone of antiviral and inflammatory responses. Their coordinated upregulation further supports the presence of an interferon-amplified activation state in T cells prior to irAE onset, a pattern that has been repeatedly associated with severe or early irAEs in previous studies (Chen et al., 2023; Khan et al., 2025). These findings suggest that T cells in irAE-susceptible individuals exist in a primed inflammatory state that may make them more vulnerable to ICI-induced hyperactivation.

Functional enrichment based on Gene Ontology revealed that the upregulated genes were enriched in biological processes related to cellular stress responses, response to cytotoxic cytokines, regulation of lymphocyte activation, intrinsic apoptotic signaling and antiviral responses (Figure 3E). These terms collectively describe a highly activated immune-inflamed transcriptional state and further support the presence of a hyper-responsive T-cell phenotype before therapy.

To determine whether these transcriptional changes overlapped with immune-related genes, we compared differentially expressed genes with immune-related genes. 77 genes overlapped, including numerous interferon-stimulated genes and cytokine-responsive targets (Figure 3F).

To explore potential pharmacological modulators of this transcriptional state, we incorporated the dysregulated immune-related genes into the CIGS perturbation framework. Multiple compounds were identified as mimickers of the irAE-associated T-cell state, such as 9-methoxycampothecin, 10-hydroxycamptothecin, ASLAN003 and PTC299. Several other agents, including Glyoxalase I inhibitor, Flavokawain A, Basimglurant, Imirestat and Narciclasine, demonstrated strong reversal potential (Figure 3G). These candidate compounds may therefore provide therapeutic avenues for attenuating or preventing T-cell hyperactivation during irAE development.

### 3.4 NK-cell transcriptomic profiling reveals strong interferon signaling activation and immune effector priming in irAE cases

**FIGURE 4:**
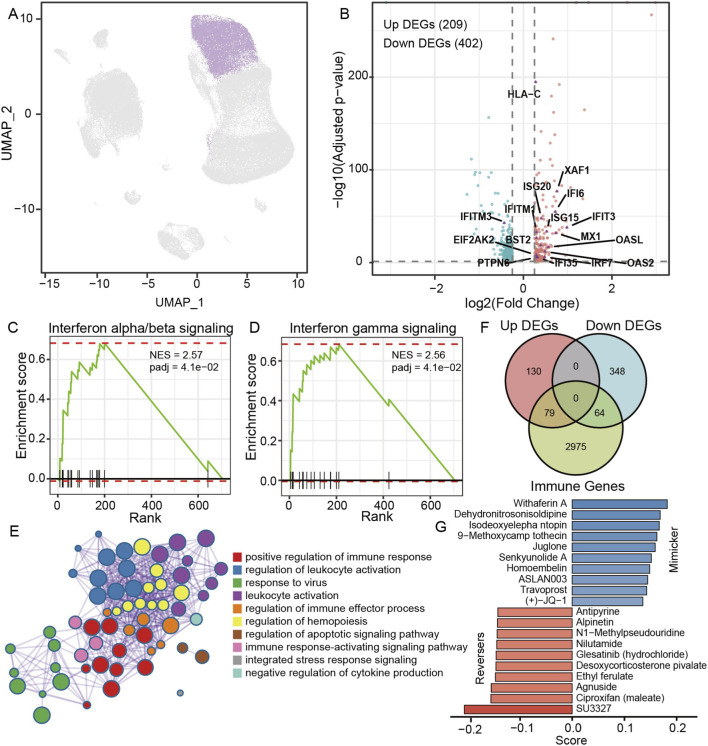
NK-cell specific transcriptional alterations reveal strong interferon activation and immune-effector priming in irAE samples. **(A)** UMAP visualization of NK cells isolated from NSCLC PBMC scRNA-seq data. **(B)** Volcano plot depicting differentially expressed genes (DEGs) between irAE and non-irAE NK cells. **(C,D)** GSEA showing significant enrichment of interferon-α/β signaling (C; NES = 2.57) and interferon-γ signaling (D; NES = 2.56) in irAE NK cells. **(E)** Venn diagram illustrating the overlap between NK-cell DEGs and immune-related genes. **(F)** Functional enrichment network of upregulated genes in irAE NK cells. **(G)** Perturbation-based compound screening using the CIGS database.

To further examine the contribution of innate lymphocytes to irAE pathogenesis, we isolated all NK-cell populations from the NSCLC single-cell dataset (Figure 4A) and compared their transcriptomes between irAE and non-irAE samples. Differential expression analysis identified 209 upregulated and 402 downregulated genes in irAE-associated NK cells (Figure 4B).

GSEA demonstrated strong activation of interferon-α/β signaling (NES = 2.57) and interferon-γ signaling (NES = 2.56) in NK cells from irAE samples (Figures 4C,D). Consistent with these pathway-level enrichments, most of the core interferon-stimulated genes were also present among the upregulated differentially expressed genes in irAE-associated NK cells (Figure 4B). These included MX1, ISG15, IFIT1, IFIT3, IFI6, OASL, OAS2 and XAF1, all of which represent canonical ISG markers that form the transcriptional backbone of type I and type II interferon responses. This mirrored the transcriptional features observed in irAE-associated T cells, suggesting a coordinated interferon-driven activation program across both innate and adaptive lymphocyte compartments in patients who later develop irAEs.

Functional enrichment analysis further showed that upregulated genes were strongly associated with positive regulation of immune response, leukocyte activation, immune effector processes, apoptotic signaling, hemopoiesis and integrated stress-response pathways (Figure 4E). These findings indicate that NK cells in irAE-prone individuals exist in a highly primed activation state prior to ICI initiation.

Integration of NK-cell differentially expressed genes with immune-related gene sets identified 79 upregulated and 64 downregulated immune genes (Figure 4F), supporting the convergence of NK-cell dysregulation on canonical inflammatory and interferon signaling pathways.

To identify compounds capable of modulating this irAE-specific NK-cell program, we queried the dysregulated immune-related genes against the CIGS database (Figure 4G). Several traditional Chinese medicine–derived molecules emerged as NK-cell mimickers, including Isodeoxyelephantopin, 9-methoxycampothecin (Camptothecin derivatives), Juglone and Homoembelin. Some compounds show strong mechanistic consistency with the interferon-amplified NK-cell phenotype observed in irAE samples. Sesquiterpene lactones such as Isodeoxyelephantopin can activate NF-κB and MAPK inflammatory pathways (Han et al., 2020; Mehmood and Muanprasat, 2022) and thus may indirectly promote interferon-stimulated gene induction. Camptothecin derivatives, for example, via low-dose topoisomerase I inhibition, can trigger DNA-damage responses that activate the cGAS–STING–type I interferon axis (Pepin et al., 2017). Together, these data demonstrate that these compounds recreate cellular states involving DNA-damage signaling and interferon response activation, closely paralleling the transcriptional features of irAE NK cells. Conversely, several compounds including Antipyrine, Alpinetin, Nilutamide, Desoxycorticosterone pivalate, Ethyl ferulate, Agnuside and Ciproxifan were predicted to reverse the NK-cell transcriptional state associated with irAEs. Many of these agents are known to dampen interferon signaling or inflammatory cytokine responses, suggesting their potential to attenuate NK-cell hyperactivation and reduce irAE risk.

Collectively, these results indicate that NK cells in irAE-prone patients exhibit a pre-existing interferon-amplified and immune-effector–primed state before therapy. This heightened innate lymphocyte activation may synergize with immune checkpoint blockade, lowering the threshold for excessive inflammatory activation and thereby contributing to irAE development.

### 3.5 Experimental validation of interferon-γ signaling suppression by candidate reversal compounds

**FIGURE 5:**
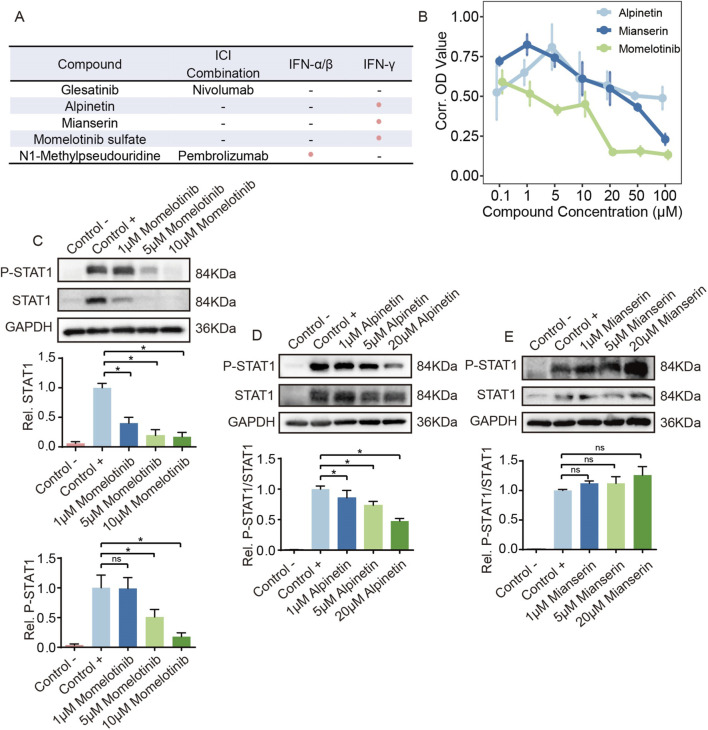
Experimental validation of interferon-γ signaling suppression by candidate reversal compounds. **(A)** Overview of candidate reversal compounds identified through the CIGS analysis. **(B)** Cell viability assessment of Jurkat T cells treated with alpinetin, mianserin, or momelotinib sulfate. Data are presented as mean ± SD. **(C)** Western blot analysis of total STAT1 and p-STAT1 protein levels in Jurkat cells treated with momelotinib. **(D)** Western blot analysis of total STAT1 and p-STAT1 protein levels in Jurkat cells treated with alpinetin. **(E)** Western blot analysis of total STAT1 and p-STAT1 protein levels in Jurkat cells treated with mianserin.

To elucidate the mechanistic basis underlying the candidate compounds identified as potential irAE reversers, we systematically examined their known immunoregulatory activities using published perturbation evidence. Among these compounds, the MET/SMO inhibitor glesatinib has been clinically evaluated in combination with the PD-1 inhibitor nivolumab in advanced NSCLC (Nemunaitis et al., 2017), while N1-methylpseudouridine represents a key nucleoside modification incorporated into several mRNA-based cancer vaccines currently being tested alongside PD-1/PD-L1 blockade (Weber et al., 2024; Meade and Garvey, 2025; Pan et al., 2024) (Figure 5A).

Notably, some candidate reversal compounds are linked to the regulation of interferon-α/β and interferon-γ signaling, two pathways that were strongly activated in both T cells and NK cells from irAE-prone patients in our analysis (Figure 5A). Alpinetin, isolated from *Amomum tsao-ko*, inhibits TLR4-dependent NF-κB and MAPK signaling and suppresses pro-inflammatory cytokines including IFN-γ *in vivo*, thereby attenuating IFN-γ-associated inflammatory responses (Huang et al., 2015; Rados et al., 2024; Liu et al., 2024). Mianserin has been shown to reduce T-cell-derived IFN-γ while increasing IL-10 and TGF-β in stimulated PBMCs, shifting the cytokine milieu toward an anti-inflammatory profile (Szuster-Ciesielska et al., 2003). Momelotinib sulfate, a selective JAK2 inhibitor, directly targets the IFNGR–JAK2–STAT1 axis and suppresses downstream IFN-γ-driven inflammatory and stemness programs (Cherng et al., 2021). N1-methylpseudouridine, a widely used nucleoside modification in mRNA therapeutics, attenuates innate RNA sensing through innate immune sensors such as TLR7/8 and RIG-I, thereby reducing type I IFN production and interferon-stimulated gene induction (Nance and Meier, 2021). We next performed *in vitro* validation and mechanistic analysis of the three interferon-γ–inhibitory compounds using the Jurkat T-cell line.

Jurkat cells were used as an experimental model to systematically evaluate the effects of alpinetin, mianserin, and momelotinib on cell viability using a CCK-8 assay. As shown in Figure 5B, treatment with these compounds at low to moderate concentrations (0.1–10 μM) exerted minimal effects on Jurkat cell viability, with overall cell survival remaining largely stable. 1 n contrast, exposure to higher concentrations (≥20 μM), particularly in the momelotinib-treated group, resulted in a pronounced reduction in cell viability, indicating that high-dose treatment may impair cell survival. Based on these observations, and to exclude potential confounding effects of cytotoxicity on signaling analyses, concentration ranges without overt cytotoxicity were selected for subsequent mechanistic studies. Accordingly, momelotinib was applied at 1, 5 and 10 μM, whereas alpinetin and mianserin were applied at 1, 5, and 20 μM for Western blot analysis of key components in the interferon-γ signaling pathway.

Western blot analysis demonstrated that momelotinib sulfate reduced total STAT1 protein levels in a concentration-dependent manner (Figure 5C). Given that momelotinib is a well-characterized JAK1/2 inhibitor, this finding suggests that inhibition of upstream JAK activity leads to altered downstream STAT1 protein homeostasis, thereby modulating interferon-γ signaling. In contrast, alpinetin markedly decreased STAT1 phosphorylation without significantly affecting total STAT1 protein expression (Figure 5D), indicating that alpinetin primarily attenuates interferon-γ signaling at the level of STAT1 activation. Notably, mianserin did not induce significant changes in either STAT1 protein abundance or STAT1 phosphorylation (Figure 5E).

Collectively, these results indicate that alpinetin and momelotinib suppress interferon-γ signaling through distinct mechanisms. Alpinetin predominantly interferes with STAT1 phosphorylation and activation, whereas momelotinib sulfate modulates the pathway by inhibiting JAK1/2 activity and subsequently reducing STAT1 protein levels.

## 4 Discussion

In this study, we integrated bulk and single-cell transcriptomic data to systematically investigate the mechanisms underlying irAEs and to identify potential strategies for predicting and mitigating their occurrence. By quantifying immune infiltration signatures in pre-treatment PBMCs, we constructed machine learning models that showed promising performance in stratifying irAE risk, with Random Forest achieving the highest performance. SHAP analysis revealed that activated T-cell and NK-cell signatures were the dominant contributors to irAE susceptibility, suggesting that irAE-prone individuals harbor a pre-existing, immune-primed state before ICI therapy.

Single-cell analysis of NSCLC PBMCs further uncovered two irAE-enriched myeloid clusters exhibiting “lung-like” inflammatory transcriptional programs, characterized by enrichment of lung classical monocyte, lung dendritic cell, and bone marrow neutrophil signatures. These findings indicate that irAE-susceptible patients may possess a baseline inflammatory myeloid program potentially poised for tissue migration and immune activation. Within lymphocyte compartments, both T cells and NK cells from irAE patients showed striking upregulation of interferon-stimulated genes and strong enrichment of type I and type II interferon signaling pathways, revealing a coordinated interferon-amplified activation state that may predispose patients to ICI-induced hyperinflammation.

From a translational perspective, our findings support the potential value of PBMC-derived immune features as predictive biomarkers for irAE risk stratification. Because PBMCs can be obtained before treatment in a minimally invasive manner, baseline immune profiling may provide a practical framework for identifying patients at higher risk of toxicity before ICI initiation. Although the current predictive models are still preliminary, the overall results suggest that pre-treatment immune infiltration signatures may serve as a useful starting point for biomarker development and future clinical risk assessment.

Importantly, our perturbation-based drug screening analysis identified multiple candidate compounds capable of reversing these interferon-amplified transcriptional states. To move beyond computational prediction, we further performed *in vitro* validation and mechanistic dissection using Jurkat T cells. These experiments demonstrated that alpinetin and momelotinib suppress interferon-γ signaling through distinct molecular mechanisms: alpinetin primarily inhibited STAT1 phosphorylation, whereas momelotinib reduced total STAT1 protein levels via upstream JAK1/2 inhibition. These results provide preliminary experimental support for our *in silico* predictions and underscore that interferon signaling can be modulated at multiple regulatory nodes, offering flexibility for therapeutic intervention. However, the translational readiness of these two compounds differs substantially. Momelotinib has the clearest clinical feasibility, whereas alpinetin remains predominantly at the preclinical stage. Accordingly, their safety, pharmacology, and potential effects on antitumor immunity in the setting of irAE prevention require systematic evaluation before clinical application.

Several limitations of this study should be acknowledged. The machine learning models showed promising performance in stratifying patients at risk of developing irAEs. But the relatively small sample size of the current study, particularly the limited number of irAE cases, may increase the risk of overfitting and constrain the robustness and generalizability of the predictive models. Therefore, the observed predictive performance should be regarded as preliminary evidence of the potential utility of baseline immune infiltration patterns for irAE risk assessment, rather than as a definitive clinical prediction framework. Future studies with larger, independent, and prospectively collected cohorts will be necessary to further validate the stability, reproducibility, and clinical applicability of these models. Additionally, although Jurkat cells provided a convenient *in vitro* system for preliminary validation of interferon-γ related signaling modulation, they are an immortalized leukemia-derived T-cell line and therefore cannot fully represent the complexity of systemic immune responses in patient-derived PBMCs. Accordingly, the experimental results should be interpreted as initial mechanistic evidence rather than definitive validation of the immune processes underlying irAE development. Future studies using primary PBMCs, co-culture systems, or *in vivo* models will be necessary to further confirm the biological relevance of these findings.

Our study not only provides a conceptual framework for the early identification of patients at high risk for irAEs, but also proposes candidate compounds capable of modulating interferon signaling pathways. Future studies integrating longitudinal sampling and *in vivo* validation will be essential to translate these findings into clinically feasible strategies, with the ultimate goal of reducing irAE risk while preserving antitumor efficacy.

## Data Availability

The original contributions presented in the study are included in the article, further inquiries can be directed to the corresponding authors..
